# Exploiting Small Leakages in Masks to Turn a Second-Order Attack into a First-Order Attack and Improved Rotating Substitution Box Masking with Linear Code Cosets

**DOI:** 10.1155/2015/743618

**Published:** 2015-09-28

**Authors:** Alexander DeTrano, Naghmeh Karimi, Ramesh Karri, Xiaofei Guo, Claude Carlet, Sylvain Guilley

**Affiliations:** ^1^New York University, New York, NY 10012, USA; ^2^Rutgers University, New Brunswick, NJ 08901, USA; ^3^Security Center of Excellence, Intel Corporation, Hillsboro, OR 97124, USA; ^4^Paris 8 University, 93526 Saint-Denis, France; ^5^Télécom ParisTech, 75634 Paris, France; ^6^Secure-IC S.A.S., 35510 Cesson-Sévigné, France

## Abstract

Masking countermeasures, used to thwart side-channel attacks, have been shown to be vulnerable to mask-extraction attacks. State-of-the-art mask-extraction attacks on the Advanced Encryption Standard (AES) algorithm target S-Box recomputation schemes but have not been applied to scenarios where S-Boxes are precomputed offline. We propose an attack targeting precomputed S-Boxes stored in nonvolatile memory. Our attack targets AES implemented in software protected by a low entropy masking scheme and recovers the masks with 91% success rate. Recovering the secret key requires fewer power traces (in fact, by at least two orders of magnitude) compared to a classical second-order attack. Moreover, we show that this attack remains viable in a noisy environment or with a reduced number of leakage points. Eventually, we specify a method to enhance the countermeasure by selecting a suitable coset of the masks set.

## 1. Introduction

Traditionally, a cryptographic algorithm was considered secure if it withstood classical linear and differential cryptanalysis. A side-channel attack exploits physical characteristics of a device in order to recover secret information, such as the encryption key. Power dissipation and electromagnetic (EM) emanation side-channel attacks are of particular concern because of their low implementation cost, ease of use, and effectiveness in extracting secret information [1]. Power analysis attacks work because the amount of power (or EM emanations) dissipated by a device is dependent on the data being processed. The Advanced Encryption Standard (AES) is the standard symmetric key encryption specified by the National Institute of Standards and Technology (NIST) [2] and is also included in ISO/IEC 18033-3:2010 [3]. It is widely used in electronic systems such as automated teller machines, telecommunications, and virtual private networks. Traditional cryptanalysis cannot break AES. However, if AES is not carefully implemented, side-channel attacks can leak the secret key [1, 4–8].

### 1.1. Related Work

Masking variables is a well-known countermeasure [9–12] to protect against side-channel attacks. Sensitive variables are concealed by random variables. Masking comes in a variety of flavors; however, we consider only the* Boolean* type in this paper. Boolean masking splits a sensitive variable *x* into a number (*d* + 1) of shares by the exclusive-or (XOR) operation *x* = *x*_0_ ⊕ ⋯⊕*x*_*d*_. Each share is processed independently so that the measured leakage depends on some random value, rather than the sensitive information. A first-order masking scheme uses one mask, whereas a *d*th-order masking scheme uses *d* masks. A (*d* + 1)th-order attack targets the manipulation of *d* + 1 manipulated variables that jointly depend on a secret value. A *d*th-order masking scheme can be broken by a (*d* + 1)th-order attack [13]. Masking strategies can also be classified according to the amount of entropy used; intuitively, the more the entropy in the set of masks is, the more secure the implementations are against side-channel analysis.* Full Entropy Masking Schemes* (FEMS) draw masks from the entire mask set to conceal sensitive information [14]. In the case of AES, each plaintext byte is masked, and so each mask can take on all 256 values from *𝔽*_2_^8^.* Low Entropy Masking Schemes* (LEMS) instead draw masks from a reduced mask set, a strict subset of *𝔽*_2_^8^ [14, 15].

Masking the nonlinear portions of AES, that is, the substitution boxes (S-Boxes), can be costly. The masked S-Boxes can be calculated on the fly for each encryption [9], securely precomputed before encryption begins [16], or generated offline and stored in Read-Only Memory (ROM) or in Random Access Memory (RAM) [17]. The S-Box precomputation scheme suits AES, because the 16 S-Boxes are the same (unlike, e.g., the Data Encryption Standard—DES). However, the S-Box precomputation method significantly increases total encryption time. The masked S-Box is typically recalculated for every encryption and this S-Box recomputation can be as long as the entire AES operation, if not longer. For instance, the authors in [13] describe AES implementation that takes twice as long to encrypt a plaintext versus the equivalent unprotected version; 33% of the runtime is spent calculating the masked S-Box. The frequent reuse of the mask during the S-Box precomputation allows for horizontal attacks (deemed horizontal because multiple points along a single power trace are analyzed [18]), which exploit the high multiplicity of samples (namely, 256) to recover the mask [19–21].

Computing offline the entire set of masked S-Boxes (256 for FEMS) alleviates the extra runtime issue of S-Box recomputation but requires at least 64 kilobytes of memory which is beyond the capacity of embedded systems such as smartcards. LEMS offers a tradeoff between complexity and security. The space required for a LEMS using 16 masks out of 256 masks is that needed to store 16 S-Boxes (namely, 4 kilobytes of storage). Removing the need for lengthy masked S-Box precomputation, we notice that LEMS are less prone to attacks such as those described in [19–21]. Additional masks (as in high-order masking schemes) increase the complexity and area overhead of the design, since these extra masks have to be stored in memory or calculated at some point in time. Therefore, first-order masking schemes are the mainstream protection.

### 1.2. Contribution and Outline

Efficient first-order masking schemes (FEMS using S-Box precomputation or LEMS such as Rotating S-Box Masking [17]) reuse the same mask several times, typically at each S-Box call; therefore, a horizontal power analysis attack on 16 leakage points can reveal the mask. We show that the state-of-the-art mask-extraction attack [20] on S-Box precomputation can be retargeted towards masked AES implementation. Indeed, the attack presented in [19–21] is the core idea of this paper. At the time of writing, a similar attack was published on the DPA Contest website [22] by Nakai et al. We want to stress that both works were performed independently of each other. We therefore add value by exploring the attack parameters in order to gain a deeper understanding of the strength of the attack. This paper has three main contributions. First, we show that the attack can succeed even in the presence of noise: tiny information on the mask can be extracted, enabling a first-order attack in a second pass. Second, we find that this type of attack outperforms a classical second-order attack with respect to number of traces needed to recover the key. Third, we explore improvements of the code employed for masks of the Rotating S-Box Masking countermeasure to make the exploitation of the leakage more difficult.

The rest of the paper is organized as follows.  Section 2 proposes the mask recovery attack and validates it using publicly available data.  Section 3 discusses the attack results and attack parameters, compares the attack with a state-of-the-art second-order attack [23] in noisy environments, and proposes a countermeasure.  Section 4 concludes the paper and opens some perspectives. The Appendix exhibits a constant Hamming weight code, but with resistance against only first-order attacks. The countermeasure presented in Section 3 and the tradeoff discussed in the Appendix are two noticeable contributions with respect to the preliminary conference version of this paper [24].

## 2. The Proposed Mask Recovery Attack

We describe the implemented countermeasure, power analysis, and the proposed attack.

### 2.1. Rotating S-Box Masking

**Figure 1:**
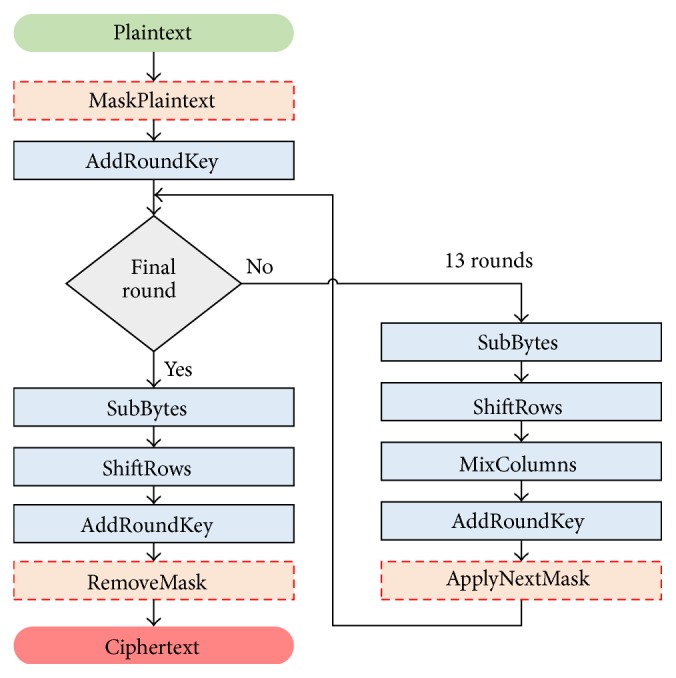
AES-256 with the Rotating S-Box Masking (RSM) protection. RSM is a Low Entropy Masking Scheme. The dashed boxes represent the operations added by RSM to AES.

A first-order masking countermeasure called* Rotating S-Box Masking* (RSM) [17] is shown in Figure 1. The dotted boxes represent the additional steps added to AES-256 by RSM. RSM is a Boolean-additive LEMS and uses a total of 16 public-knowledge masks, *m*_0−15_ ∈ *ℳ* ⊂ *𝔽*_2_^8^, one for each byte of plaintext. At the start of each encryption, a random offset *j* ∈ [0 ⋯ 15] is drawn. The offset can be thought of as the number of positions to cyclically left-rotate the base set of masks, *ℳ*_0_. The set of masks with offset *j* is denoted by *ℳ*_*j*_; for example, if the offset *j* = 0, then the masks are deployed in the following order: *ℳ*_0_ = *m*_0_, *m*_1_, *m*_2_,…, *m*_14_, *m*_15_. Thus, only 16 possibilities exist for the order of the masks, since a shift greater than 15 simply wraps the set of masks around. The masks are then XORed with the plaintext, and this result is XORed with the first round key. The S-Box is replaced by 16 masked S-Boxes, where each S-Box corresponds to an offset. This avoids the penalty of the lengthy S-Box recomputation that other masking schemes utilize (except masking schemes with S-Box secure calculation [10, 12]).* ShiftRows* is unchanged since the underlying data is not modified. The* MixColumns* operation is a special masked version. Afterwards, the next-round masks are applied while simultaneously removing the current-round masks, and the offset value is incremented. It is important to stress that the data never appear unmasked.

Interestingly, an optimization of RSM in terms of speed has been published in 2014 [25]. In this paper, we study the genuine RSM, as implemented in the DPA Contest V4 [22].

### 2.2. Power Analysis

A generic power (or EM) analysis attack has the following five steps [13]:

(1) Measure the power consumption (or EM) of a device as it encrypts (resp., decrypts) a number of plaintexts (resp., ciphertexts): we used EM traces provided by the DPA Contest V4 [22], as detailed in Section 2.3.
(2) Choose an intermediate result of the target algorithm to attack: normally, a part of the algorithm that operates on the key is attacked. However, we wish first to recover the used masks (of course, the masks set is public, but not the order in which they are used), so we target the loading of the masks, as described in Section 2.5.
(3) Calculate the intermediate results for all secret hypotheses: in this case, there are 16 possibilities for the mask set, shown in matrix **M** in Section 2.6.
(4) Apply a hypothetical power model to the calculated intermediate results: we used the Hamming weight power model, as described in Section 2.6.
(5) Compare the measured power consumption to the hypothetical power consumption to determine the secret key (or a small part of the key): this is explained in more detail in Section 2.6.

This attack is performed in two stages: (1) the preprocessing mask recovery stage and (2) CPA attack to recover the key. The basic idea is to recover an estimate of the masks from each power trace and then launch a horizontal (attacking many samples from a single trace) CPA attack against the 16 possible combinations of the mask. Recovering the masks allows us to undo the countermeasure so that we can correctly predict some intermediate value, for example, the S-Box output. Thus, a second CPA attack, vertical (attacking the same time instance across many traces) this time, reveals the key. Both stages are first-order attacks.

### 2.3. Experimental Setup

The AES-256 RSM is implemented on an Atmel ATMega-163 smartcard connected to a SASEBO-W board [22]. EM traces were captured using a Langer EM near-field probe RF-U 5-2, sampled at 500 MS/s by a Lecroy Waverunner 6100A oscilloscope.

### 2.4. Leakage Detection

In order to attack efficiently, it is important to precisely locate the leaking samples in the traces: this is the purpose of the leakage detection phase.

**Figure 2:**
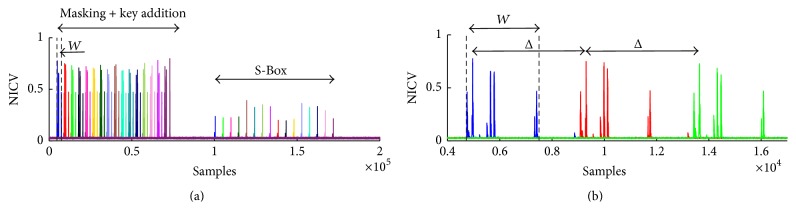
NICV for each plaintext byte over 10,000 traces. (a) AES operations are identifiable. (b) NICV for the first 3 bytes of plaintext. Each byte exhibits similar characteristics, which implies the operation taking place a number times, but each time processing different data.

We use Normalized Interclass Variance (NICV) [26], which is an analysis of variance (ANOVA) *F*-test, to identify leakage in power traces. The NICV relies on publicly available information (such as known plaintexts or ciphertexts). Let *T* be the set of power traces and let *X* be the corresponding set of plaintext bytes. The NICV is calculated as NICV = Var(**E**[*T*∣*X*])/Var(*T*), where **E** is the expectation operator, Var is the variance operator, and 0 ⩽ |NICV| ⩽ 1. It is thus a normalized indicator of leakage, which does not require the knowledge of the key.  Figure 2 shows the NICV calculated for each plaintext byte using 10,000 traces and reveals useful information to the attacker. With knowledge of the algorithm, he/she can distinguish when different operations take place. The 16 peaks in Figure 2(a) from samples 0 to 75,000 suggest the* AddRoundKey* operation, while the second set of 16 peaks beginning at sample point 10^5^ signifies the* SubBytes* operation. An attacker can use this knowledge to extract leakage samples that belong to a certain operation.

The attacker now has a rough idea of the time frame when each operation takes place and can even determine the amount of time to process each byte by examining Δ, the distance between the peaks in Figure 2(b). Figure 2(a) shows that each plaintext byte is operated on only once before it enters the S-Box; that is, there is only one time interval when leakage occurs for each plaintext byte before the S-Box. Therefore, the plaintext loading, masking operation, and* AddRoundKey* must all take place within the same time interval. Moreover, the order and morphology of each NICV curve tell the attacker that the same set of operations is applied 16 times in a row, beginning with byte 0 and ending with byte 15. Consequently, the attacker now has an idea about the mask order.

### 2.5. Extract Leaky Samples

The attacker then chooses a window *W* of width Δ and extracts possible candidates for the time samples when each mask is loaded. The attacker can use the NICV (or some other leakage detection tool [26] such as Sum-of-Square Differences (SOSD) or Sum-of-Square *t*-test (SOST)) to minimize the amount of points he/she will attack by considering only leakage measurements above a certain threshold (determined empirically), or he/she can simply attack every point in the window. The attacker selects *τ* samples to attack from a single power trace and stores their leakage measurements, *v*, into the first column of the *τ* × 16 matrix **V**. Each column of **V** is then filled in by extracting the leakage measurement located exactly Δ samples further from the previous measurement:

(1) $$\begin{array}{c} V=\left[\begin{array}{ccccc} t_{0} & t_{0}+\Delta & t_{0}+2\Delta & \cdots & t_{0}+15\Delta \\ t_{1} & t_{1}+\Delta & t_{1}+2\Delta & \cdots & t_{1}+15\Delta \\ \vdots & \vdots & \vdots & \ddots & \vdots \\ t_{\tau -1} & t_{\tau -1}+\Delta & t_{\tau -1}+2\Delta & \cdots & t_{\tau -1}+15\Delta \end{array}\right]. \end{array}$$

### 2.6. Recover the Mask Offset

The next step is to launch a modified CPA attack on the subtraces in **V**. Since we do not know in which order the masks were loaded, we guess every combination, as shown in the 16 × 16 matrix **M** = [*ℳ*_0_ ⋯ *ℳ*_15_]^*⊤*^. Each column of **M** corresponds to an offset applied to the base set of masks *ℳ*_0_, where

(2) $$\begin{array}{c} M=\left[\begin{array}{ccccc} m_{0} & m_{1} & m_{2} & \cdots & m_{15}\\ m_{1} & m_{2} & m_{3} & \cdots & m_{0}\\ \vdots & \vdots & \vdots & \ddots & \vdots \\ m_{15} & m_{0} & m_{1} & \cdots & m_{14} \end{array}\right]. \end{array}$$

We apply a Hamming weight power model *w*_*H*_(·) to the mask matrix **M**, which is generally a good model for microprocessors [13, 27]. The hypothetical power consumption is **H** = *w*_*H*_(**M**). The next step is to compare the modeled power consumption with the measured power consumption. If we assume the power model to be linear, for example, Hamming weight or Hamming distance, a natural choice for the attack is the correlation coefficient. Correlation power analysis (CPA) evaluates the amount of correlation between a set of measured power traces *T* and a model of the key-dependent device leakage, *L* [5], and is calculated for every time sample. Pearson's correlation coefficient is calculated as *ρ*(*T*, *L*) = cov(*T*, *L*)/(*σ*_*T*_ *σ*_*L*_); however, this can be difficult (or impossible) to compute, and so we instead use an estimate $\hat{\rho }$ (where $|\hat{\rho }|⩽1$) which is calculated as $\sum_{i=0}^{n-1}\left(t_{i}-\bar{t_{i}}\right)\left(l_{i}-\bar{l_{i}}\right)/\sqrt{\sum_{i=0}^{n-1}{\left(t_{i}-\bar{t_{i}}\right)}^{2}\sum_{i=0}^{n-1}{\left(l_{i}-\bar{l_{i}}\right)}^{2}}$ for the set of traces *T* (containing *n* traces *t*_*i*_) and hypothetical power model *L*, containing *n* hypothetical power consumption values *l*. Wrong guesses for the key will have correlations close to 0, while the correct guess will have $\left|\hat{\rho }\right|$ close to 1 (assuming the power model is accurate). We calculate $\hat{\rho }(V,H)$, which leads to 16 correlation coefficients. Each correlation coefficient corresponds to a mask offset. By choosing the location where $\max\hat{\rho }(V,H)$ occurs, we can guess the offset. The overall procedure is exhibited in Algorithm 1. Using the offset guess, we can predict the S-Box output and deploy a CPA attack to recover the key.

## 3. Results

This attack is feasible since the device leaks the Hamming weight of the masks when they are loaded from memory. Once the masks are recovered, extracting the key is straightforward. Our attack requires 10.1 traces to fully recover the key, while an attack on unprotected implementation requires 9.9 traces and can be considered as a lower bound regarding the number of traces. Our attack is close to that bound; the reason that we need slightly more traces is because we do not always correctly guess the offset. Comparing our offset guesses with the actual mask offsets, we were able to successfully guess the offset 91% of the time. Recall that the estimation error of the mean in a Bernoulli process is *p*(1 − *p*)/*n*_rep_, where *p* = 0.91 and *n*_rep_ is the number of repetitions; namely, *n*_rep_ = 10,000. The success rate is estimated over 10,000 traces with accuracy ≈10^−5^.

### 3.1. Mask Recovery Success Rate

Figure 4(a) shows the success rate of recovering the mask for various signal-to-noise ratios (SNRs). The probability of correctly guessing the offset at random is 1/16, or 6.25%: we exceed this value for all SNRs >2^5^ (i.e., *σ*_noise_ > 30). Therefore, using our method is preferred for naively guessing for most noise levels.

### 3.2. Tweaking the Algorithm Parameters

**Figure 3:**
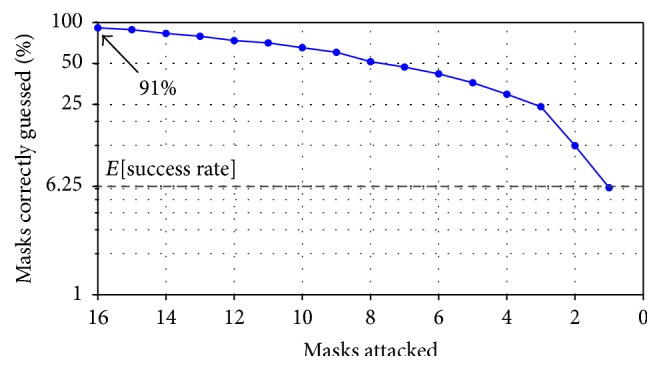
Mask recovery success rate as a function of number of masks attacked.

We examine how the algorithm parameters affect the mask recovery success rate. If only one mask (out of a possible 16) is attacked, the success rate equates to the expected value for naively guessing the mask. Indeed, with 1 mask, there is no “rotation” possible; hence, the mask is “horizontally indistinguishable.” Thus, an attacker gains no advantage by trying to recover the mask by attacking only one sample, since the extra computation time does not lead to an increase in success rate. However, attacking 2 masks, that is, {*m*_0_, *m*_1_}, allows the pair to be distinguished with 11% success rate, slightly outperforming naive guessing. As shown in Figure 3, the success rate increases linearly as the number of masks increases, demonstrating the positive relationship between mask entropy and number of masks attacked.

The attacker can also vary the width of the window where he/she suspects the masking operation to occur. Enlarging the window linearly increases the computational effort; that is, increasing the width by *n* samples leads to an attack complexity of *𝒪*(*n*). Compare this to a second-order attack, where an increase in *n* samples requires *n*(*n* − 1)/2 calculations [28], or complexity *𝒪*(*n* ^2^).

### 3.3. Comparison with State of the Art in the Presence of Noise

Noise increases the difficulty of carrying out a successful power attack; that is, an attacker is required to measure more power traces. Common sources of noise include electronic noise from other circuit components, measurement errors, and clock jitter [13, 27]. Most of the noise in cryptographic devices can be approximated by a normal distribution ~*𝒩*(0, *σ* ^2^) [13]. In order to determine the influence of noise on our attack, we artificially corrupt the power traces by introducing additive white Gaussian noise ~*𝒩*(0, *σ* ^2^).

We compare our attack with a state-of-the-art second-order attack, namely, the bivariate attack, using a centered product as combination function in [23]. This type of attack is ideal for first-order masking schemes implemented in software and was proven to be optimal in the presence of noise [23].

**Figure 4:**
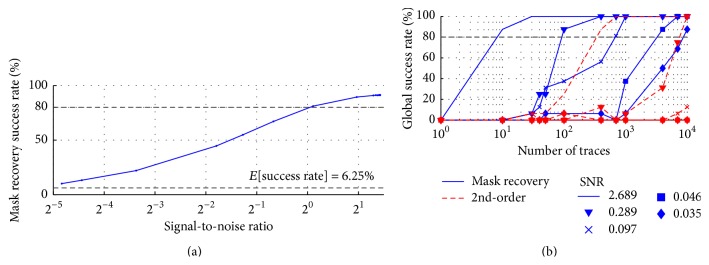
(a) Mask recovery success rate for 10,000 traces. (b) Global success rate (GSR) versus number of traces for different noise levels. The mask recovery attack outperforms the second-order attack by at least two orders of magnitude at every SNR.

Figure 4(b) shows the evolution of global success rate (GSR) as a function of number of traces attacked and signal-to-noise ratio (SNR). GSR is the probability to recover the full key. We define an attack as being successful if GSR⩾80%; conversely, we define a failed attack if the GSR fails to reach 80% within 10,000 traces. The best-case attack scenario is SNR = 2.689; that is, no artificial noise is added. The best-case mask recovery attack requires 10 traces to succeed, whereas the best-case second-order attack does not succeed until 300 traces. The mask recovery attack is more resilient to noise since, for a given number of power traces, the success rate will be higher for all SNRs. Regardless of the noise level, our mask recovery attack (empirically) reveals the key faster than a traditional bivariate attack.

The mask recovery attack outperforms the second-order attack by about two orders of magnitude for SNR⩾0.289. The second-order attack fails for SNR < 0.289, whereas the mask recovery attack succeeds for 0.035 ⩽ SNR ⩽ 2.689. The lower performance of the second-order attack can be attributed to the leakage combination function. Indeed, by combining multiple leakages, the noise is amplified [23]. By choosing an optimal prediction function, the noise amplification can be minimized, but much more traces must be analyzed for a successful attack as shown in Figure 4(b).

### 3.4. How to Defend against this Attack?

The mask set *ℳ* is a linear code of parameters [8,4, 4] and of weights enumerator polynomial *X* ^8^ + 14*X* ^4^ *Y* ^4^ + *Y* ^8^, which means that one codeword has a Hamming weight of 0, another one has a Hamming weight of 8, and the remaining 14 have Hamming weights of 4. One possible solution to thwart this attack is to generate all the masks with the same Hamming weight (called constant-weight codes). In this case, every column in the hypothetical power matrix **H** would be identical. If this* constant-weight code* strategy is applied, the designer must carefully consider which masks are chosen, so that the amount of leaked information is minimized. The* constant-weight code* strategy can defend against our attack and against first-order attacks only. No set of constant-weight code masks can defend against second-order (or higher) attacks as proved in the Appendix. This only applies to 8-bit software implementation, that is, a typical smartcard; we did not consider other architectures.

The* constant-weight code* strategy assumes all bits in a computer word leak equally, which is not realistic. Thus, we propose an alternative countermeasure that requires no extra resources, defends against mask-recovery attacks, and provides the same protection against first-order attacks as plain RSM. The strategy consists in (approximately) balancing the Hamming weights of the codewords belonging to *ℳ*. It has been proven in [29] that all the cosets *y* ⊕ *ℳ* (for *y* ∈ *𝔽*_2_^8^) of the studied code *ℳ* provide the same level of security, regarding monovariate attacks. Three options exist for the weight distribution. The probability that a randomly chosen element of the code has Hamming weight *h* is given below, for *h* ∈ ⟦0,8⟧:

(1) (1/16, 0,0, 0, 14/16, 0,0, 0, 1/16) if *y* ∈ *ℳ*.
(2) (0, 1/16, 0, 7/16, 0, 7/16, 0, 1/16, 0) if there is one codeword of weight 1 in *y* ⊕ *ℳ*.
(3) (0,0, 4/16, 0, 8/16, 0, 4/16, 0,0) if there is one codeword of weight 2 in *y* ⊕ *ℳ*.

**Figure 5:**
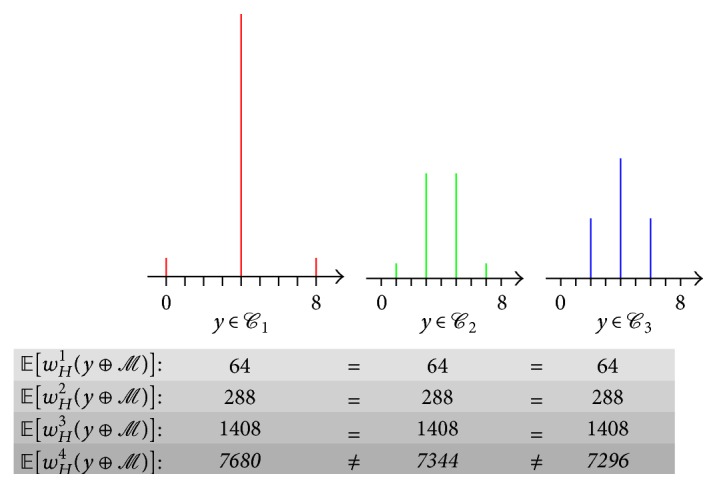
Distribution *h* ∈ ⟦0,8⟧ of the Hamming weights of the cosets *y* ⊕ *ℳ* of the RSM code *ℳ*.

This means that *𝔽*_2_^8^ can be partitioned in three partitions: *𝒞*_1_, *𝒞*_2_, and *𝒞*_3_. The distribution of *w*_*H*_(*y* ⊕ *ℳ*) is given in Figure 5, along with some noncentral moments (of degrees 1, 2, 3, and 4).

Now, by the property of the code, the variance of the Hamming weights is the same in those three cases. Namely, it is equal to 2. Indeed, the expectation of the Hamming weights is 4 in all four cases. Thus, the expectation of the square of the centered Hamming weights is, respectively, equal to

(3) 2=116×−42+1416×0+116×42=116×−32+716×−12+716×12+116×32=416×−22+816×0+416×22.

Still, it is clear that if there is a leakage in “SPA” (Simple Power Analysis), then it is more advantageous to use the code such that the Hamming weight distribution is taking only values 2, 4, and 6. So, for instance, an improvement can be obtained by using

(4) M′=0x02,0x0d,0x34,0x3b,0x51,0x5e,0x67,0x68,0x97,0x98,0xa1,0xae,0xc4,0xcb,0xf2,0xfd

instead of

(5) M=0x00,0x0f,0x36,0x39,0x53,0x5c,0x65,0x6a,0x95,0x9a,0xa3,0xac,0xc6,0xc9,0xf0,0xff.

The variance of the code has not changed, only the amplitude of the patterns. Whereas the original code had a range of amplitudes from 0 to 8, the new code has a range from 2 to 6. Thus, in the presence of noise, the SNR is reduced by 50%, making it more difficult to recover the mask.

This is reflected in Figure 5 by the new proposed* affine* code ${ℳ}^{\prime }=ℳ⊕0x2$ (see (4)) having a smaller* kurtosis* (4th-degree moment) than* linear* code *ℳ* (see (5)). Reducing the first (nonzero) correlating moment is indeed the strategy of state-of-the-art side-channel attacks on masking schemes [30].

## 4. Conclusion and Perspectives

We demonstrated how to recover a set of masks used in software implementation of AES with RSM. Our attack outperforms a traditional bivariate attack by two orders of magnitude and can succeed even in heavy noise. We show how the attack parameters affect the success rate; namely, attacking just 2 (out of 16) yields a better mask recovery success rate versus naive guessing. It is not enough to say implementation is first-order (or second-order, etc.) secure. Indeed, we showed that the countermeasure that could stop our attack can only defend against traditional first-order attacks. Further avenues of research involve empirically validating the countermeasure and extending this attack to other masking schemes (including higher-order masking schemes). Besides, it is interesting to study the security gain obtained by stacking other protections, such as S-Boxes shuffling, on top of RSM. Similar directions can be found in this prospective document [31] which gives the roadmap of the forthcoming DPA Contest V4 contests.

## Figures and Tables

**Algorithm 1:**
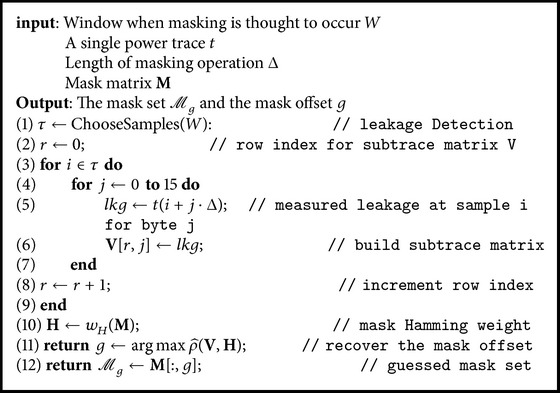
Mask recovery.

**Table 1:** Horizontally and vertically balanced code with length 8 and 16 codewords.

	Components								Sum of bits
	8	7	6	5	4	3	2	1	
Codewords	0	0	0	0	1	1	1	1	4
	1	1	1	1	0	0	0	0	4
	0	0	1	1	0	0	1	1	4
	1	1	0	0	1	1	0	0	4
	0	1	0	1	0	1	0	1	4
	1	0	1	0	1	0	1	0	4
	0	1	1	0	0	1	1	0	4
	1	0	0	1	1	0	0	1	4
	1	1	1	0	0	0	0	1	4
	0	0	0	1	1	1	1	0	4
	1	1	0	1	0	0	1	0	4
	0	0	1	0	1	1	0	1	4
	1	0	1	1	0	1	0	0	4
	0	1	0	0	1	0	1	1	4
	0	1	1	1	1	0	0	0	4
	1	0	0	0	0	1	1	1	4

Sum of bits	8	8	8	8	8	8	8	8	

## Appendix

Constant-weight codes are codes where all codewords share the same Hamming weight. They are also called *m* of *n* codes. Of particular interest are balanced codes, introduced by Knuth in 1986 [32], since they fit the basic requirement of masking. A special case for codes of length *n* = 8 is 6b/8b codes [33], used in serial communication lines to maintain DC balance in a communications system. However, in this 6b/8b code, there are 64 codewords, which is too large. Our requirements on the code can be summarized as follows: (1) the codewords must all have the same weight; (2) the code must have a large dual distance (see requirement explained in [34, 35]); (3) the code must have a size less than or equal to 16 (the number of AES substitution boxes). Nonzero balanced codes are nonlinear. Indeed, a linear code contains the null vector. Thus, the codewords have zero weight, and so the only linear balanced code is {0}.

Care must be taken that, in this appendix, “*balanced*” can have two meanings depending on the context:

Horizontally: it can be that each codeword contains an equal number of zero and one bits.
Vertically: each component (or tuples of components) is represented uniformly.

It is possible to find balanced codes with size two. For instance, on *n* = 8 bits, the code made up of codewords (01010101)_2_ and (10101010)_2_ is balanced. This is equivalent to saying that the code has dual distance at least 2. However, its dual distance is exactly 2: it allows protection against first-order attacks and not against zero-offset second-order attacks. The pair of two components is not balanced. For example, the two least significant bits of the codewords are (01)_2_ and (10)_2_: the values (00)_2_ and (11)_2_ are missing. In fact, a code of dual distance 3 must have at least a size 4. We need first a lemma about codes of length *n* with constant weight *w*.

Lemma A.1 A.1. Let *C* be a code of length *n* and constant weight *w*. If *C* is balanced, then *n* is even and *w* = *n*/2.

Proof A balanced code is such that, for all *a* of unitary Hamming weight (*w*_*H*_(*a*) = 1), ∑_*c*∈*C*_(−1)^*a*·*c*^ = 0. Now,

(A.1) ∑a/wHa=1 ∑c∈C−1a·c∑c∈C ∑a/wHa=1−1a·c=∑c∈C ∑a/wHa=11−2a·c=∑c∈Cn−2∑a/wHa=1a·c=Cn−2w.

This sum is also equal to zero, so we have *n* = 2*w*.

We have this practically relevant result.

Proposition A.2 A.2. A constant Hamming weight binary code has dual distance strictly less than 3.

Proof Let *f* be the indicator of a constant Hamming weight code *C*. We define the Fourier transform of *f* as $\hat{f}(a)=\sum_{x}f(x){\left(-1\right)}^{a\cdot x}$. If *C* has dual distance 3, then, for all *i*, *j* = 1,…, *n*,  *i* ≠ *j*, we have $\hat{f}(e_{i}⊕e_{j})=0$, where *e*_*i*_ is the vector of Hamming weight 1 in which the only 1 is at coordinate *i*. So we have $\sum_{i=1}^{n}\hat{f}(e_{i}⊕e_{j})=\hat{f}(0)=\left|C\right|$, which is also

(A.2) $$\begin{array}{c} \underset{x\in C}{\sum }{\left(\;-1\;\right)}^{x_{j}}\overset{n}{\underset{i=1}{\sum }}{\left(\;-1\;\right)}^{x_{i}}=\underset{x\in C}{\sum }{\left(\;-1\;\right)}^{x_{j}}\left(\;n-2w_{H}\left(\;x\;\right)\;\right)=0. \end{array}$$

Therefore, *C* is empty.

Proposition A.2 says that we can have constant Hamming weight codewords, but simply with protection against first-order attacks.

Example A.3 A.3 (*n* = 8 and *w* = 4). The following (nonlinear) code has parameters (8,16,2). It has constant Hamming weight *n*/2 = 4 and is balanced (more precisely, it has dual distance 2):

(A.3) C=0x0f,0xf0,0x33,0xcc,0x55,0xaa,0x66,0x99,0xe1,0x1e,0xd2,0x2d,0xb4,0x4b,0x78,0x87.

In order to better highlight the two dimensions of balancing of this code, we represent it in Table 1 in binary and add a “*sum of bits*” column and line.

Thus, this code can protect as well against

(i) horizontal side-channel analyses, since all codewords have the same Hamming weight (namely, *n*/2 = 4 bit),
(ii) vertical side-channel analyses, since each component is statistically balanced (i.e., each bit as probability 8/16 = 1/2).

## References

[1] Kocher P. C., Jaffe J., Jun B. (1999). Differential power analysis. *Advances in Cryptology—CRYPTO ’99*.

[2] National Institute of Standards and Technology (2001). *FIPS 197 Advanced Encryption Standard*.

[3] ISO (2013). *Information Technology—Security Techniques—Encryption Algorithms—Part 3: Block Ciphers*.

[4] Kaminsky A., Kurdziel M., Radziszowski S. An overview of cryptanalysis research for the advanced encryption standard.

[5] Brier É., Christophe C., Olivier F., Joye M., Jean-Jacques Q. (2004). Correlation power analysis with a leakage model. *Cryptographic Hardware and Embedded Systems—CHES 2004*.

[6] Bogdanov A., Oswald E., Rohatgi P. (2008). Multiple-differential side-channel collision attacks on AES. *Cryptographic Hardware and Embedded Systems—CHES 2008*.

[7] Moradi A., Mischke O., Eisenbarth T., Mangard S., Standaert F.-X. (2010). Correlation-enhanced power analysis collision attack. *Cryptographic Hardware and Embedded Systems, CHES 2010*.

[8] Gierlichs B., Batina L., Tuyls P., Preneel B., Oswald E., Rohatgi P. (2008). Mutual information analysis. *Cryptographic Hardware and Embedded Systems—CHES 2008*.

[9] Prouff E., Rivain M., Kim S., Yung M., Lee H.-W. (2007). A generic method for secure sbox implementation. *Information Security Applications*.

[10] Rivain M., Prouff E., Mangard S., Standaert F.-X. (2010). Provably secure higher-order masking of AES. *Cryptographic Hardware and Embedded Systems, CHES 2010*.

[11] Prouff E., Giraud C., Aumônier S., Goubin L., Matsui M. (2006). Provably secure S-box implementation based on fourier transform. *Cryptographic Hardware and Embedded Systems—CHES 2006*.

[12] Coron J.-S., Nguyen H. Q., Oswald E. (2014). Higher order masking of look-up tables. *Advances in Cryptology—EUROCRYPT 2014*.

[13] Mangard S., Oswald E., Popp T. (2008). *Power Analysis Attacks: Revealing the Secrets of Smart Cards*.

[14] Ye X., Eisenbarth T., Francillon A., Rohatgi P. (2013). On the vulnerability of low entropy masking schemes. *Proceedings of the 12th International Conference on Smart Card Research and Advanced Applications (CARDIS ’13), November 2013*.

[15] Grosso V., Standaert F.-X., Prouff E. (2014). Low entropy masking schemes, revisited. *Smart Card Research and Advanced Applications: 12th International Conference, CARDIS 2013, Berlin, Germany, November 27–29, 2013. Revised Selected Papers*.

[16] Herbst C., Oswald E., Mangard S., Zhou J., Yung M., Bao F. (2006). An AES smart card implementation resistant to power analysis attacks. *Applied Cryptography and Network Security*.

[17] Nassar M., Souissi Y., Guilley S., Danger J.-L. RSM: a small and fast countermeasure for AES, secure against 1st and 2nd-order zero-offset SCAs.

[18] Clavier C., Feix B., Gagnerot G., Roussellet M., Verneuil V. (2010). Horizontal correlation analysis on exponentiation. *Information and Communications Security: 12th International Conference, ICICS 2010, Barcelona, Spain, December 15–17, 2010. Proceedings*.

[19] Pan J., den Hartog J. I., Lu J., Youm H. Y., Yung M. (2009). You cannot hide behind the mask: power analysis on a provably secure S-box implementation. *Information Security Applications*.

[20] Tunstall M., Whitnall C., Oswald E. (2014). Masking tables—an underestimated security risk. *Fast Software Encryption: 20th International Workshop, FSE 2013, Singapore, March 11–13, 2013. Revised Selected Papers*.

[21] Bruneau N., Guilley S., Najm Z., Teglia Y., Handschuh H., Güneysu T. (2015). Multi-variate high-order attacks of shuffled tables recomputation. *Cryptographic Hardware and Embedded Systems—CHES 2015: 17th International Workshop, Saint-Malo, France, September 13–16, 2015. Proceedings*.

[22] http://www.dpacontest.org.

[23] Prouff E., Rivain M., Bevan R. (2009). Statistical analysis of second order differential power analysis. *IEEE Transactions on Computers*.

[24] DeTrano A., Guilley S., Guo X., Karimi N., Karri R. Exploiting small leakages in masks to turn a second-order attack into a first-order attack.

[25] Yamashita N., Minematsu K., Okamura T., Tsunoo Y. A smaller and faster variant of RSM.

[26] Bhasin S., Danger J.-L., Guilley S., Najm Z. Side-channel leakage and trace compression using normalized interclass variance.

[27] Kocher P., Jaffe J., Jun B., Rohatgi P. (2011). Introduction to differential power analysis. *Journal of Cryptographic Engineering*.

[28] Oswald E., Mangard S., Herbst C., Tillich S. (2006). Practical second-order DPA attacks for masked smart card implementations of block ciphers. *Topics in Cryptology—CT-RSA 2006*.

[29] Carlet C., Guilley S. Side-channel indistinguishability. https://hal.archives-ouvertes.fr/hal-00826618.

[30] Moradi A., Standaert F.-X. (2014). Moments-correlating DPA. *IACR Cryptology ePrint Archive*.

[31] Bhasin S., Bruneau N., Danger J.-L., Guilley S., Najm Z., Chakraborty R. S., Matyas V., Schaumont P. (2014). Analysis and improvements of the DPA contest v4 implementation. *Security, Privacy, and Applied Cryptography Engineering: 4th International Conference, SPACE 2014, Pune, India, October 18–22, 2014. Proceedings*.

[32] Knuth D. E. (1986). Efficient balanced codes. *IEEE Transactions on Information Theory*.

[33] Widmer X. A. DC-balanced 6b/8b transmission code with local parity.

[34] Bhasin S., Carlet C., Guilley S. Theory of masking with codewords in hardware: low-weight dth-order correlation-immune boolean functions. http://eprint.iacr.org/2013/303.

[35] Carlet C., Guilley S. (2014). Correlation-immune Boolean functions for easing counter measures to side-channel attacks. *Algebraic Curves and Finite Fields: Cryptography and Other Applications*.

